# Dual action of herbal compounds in *Klebsiella pneumoniae* infection and associated inflammatory diseases

**DOI:** 10.3389/fimmu.2026.1767981

**Published:** 2026-02-23

**Authors:** Uzma Saqib, Sakina Ratlamwala, Nidhi Kibe, Mirza S. Baig, Krishnan Hajela, Sadhana Sharma

**Affiliations:** 1 School of Life Sciences, Devi Ahilya Vishwavidyalaya, Indore (MP), India; 2 Mehta Family School of Biosciences & Biomedical Engineering, Indian Institute of Technology Indore (IITI), Indore, India; 3 Department of Biochemistry, All India Institute of Medical Sciences, Patna, India

**Keywords:** disease, herbal compounds, infection, inflammation, *Klebsiella pneumoniae*, phytochemicals, therapeutics

## Abstract

*Klebsiella pneumoniae* (Kp) is a multidrug-resistant (MDR) pathogen responsible for severe infections such as pneumonia, sepsis, and urinary tract infections. Its pathogenicity includes both bacterial virulence factors and host-driven inflammatory responses thereby complicating treatment outcomes. Herbal compounds (phytochemicals) have recently gained attention as promising dual-action therapeutic agents that target both infection and inflammation. Phytochemicals such as curcumin, berberine, quercetin, resveratrol, and several medicinal plant extracts have demonstrated an integrated ability to mitigate both infection as well as host inflammation in preclinical studies. Their ability to attenuate virulence, reduce oxidative stress, and regulate host immune signaling positions them as potential candidates for adjunctive therapy against Kp infections. Furthermore, phytochemical-antibiotic combinations demonstrate synergistic effects, enhancing bacterial clearance and reducing antibiotic dosage requirements. Overall, the dual action of phytochemicals makes them as valuable candidates for integrative therapies against *Kp* infections and related inflammatory diseases. Unlike the prior reviews, the present review uniquely focusses the dual antibacterial and immunomodulatory actions of plant-derived compounds against Kp. It adds a novel perspective integrating the therapeutic promise of phytochemicals with a systematic analysis of their translational limitations. Further, it provides a rational framework to guide future development of phytochemicals as potent and clinically viable therapeutics against Kp infections.

## Introduction

*Klebsiella pneumoniae* (Kp), a Gram-negative opportunistic pathogen (1, 2), is a leading cause of pneumonia, sepsis, and urinary tract infections, particularly in hospitalized patients (3, 4). Infection severity is influenced by bacterial virulence along with host’s immune response (5). Recognition of bacterial components, especially lipopolysaccharide (LPS), by pattern recognition receptors such as Toll-like receptor 4 (TLR4) (6, 7), triggers downstream signaling cascades including NF-κB and Mitogen-activated protein kinase (MAPK) (8), leading to the production of pro-inflammatory cytokines (9). While this response is essential for bacterial clearance, excessive or uncontrolled activation can cause tissue damage, septic shock, and organ failure (10).

In this context, plant-derived natural compounds have emerged (11) as promising therapeutic agent (12) that act at the intersection of infection and immunity (13). **Table 1** represents the list of phytochemicals along with their class, botanical source, MIC values and synergistic effect against Kp strain and cytotoxicity values. These phytochemicals not only exert direct antibacterial effects against Kp, such as disrupting cell membranes, inhibiting biofilm formation, and interfering with bacterial enzymes, but also modulate host inflammatory pathways (101). By targeting TLR4-mediated NF-κB activation, MAPK signaling, and inflammasome responses, plant compounds alleviate inflammation (102). This integrated antibacterial and immunomodulatory action positions plant-derived molecules as potential agents in the treatment of multidrug-resistant (MDR) Kp infections (103).

**Table 1:** Phytochemicals against Klebsiella pneumoniae (Kp): chemical class, botanical source, MIC values, synergistic activity, and cytotoxicity profiles.

Antibacterial compounds	Class	Source	MIC values	Synergistic effect	Cytotoxicity IC 50 value	Reference
Allyl isothiocyanate	Organosulphur Compound	Cruciferous Vegetables like Mustard seeds, horseradish and wasabi	10- 1000 µg/ml	_	25- 50 µM	(14, 15)
Resveratrol	Stilbenoids	Berries, Peanuts and Grapes	50 - > 500 µg/ml	Enhances antimicrobial activity of polymixin B and colistin in combination with resveratrol.	> 100 µM	(16) (17) (16)
Coumarins derivatives	Phenolic Compounds	Mesua ferrea	–	_	7-35 µM	(18)
Berberine	Alkaloids	Barberry	16- 512 µg/ml	Berberine enhances the potential of rifaximin.	250 µM	(19) (20) (21)
Mahanimbine		Curry Leaves	25- 200 µg/ml	_		(22) (23)
Piperine		Black Pepper	< 100 µg/ml	_	>200 µM	(24) (25) (26)
Propolis	Flavanoids	Poplar	2- 1000 µg/ml	Enhances antimicrobial efficacy, reverses bacterial resistance, and permits lower drug doses.		(27) (28) (29)
Quercetin		Apples, Onion, Spinach and Tea	16–256 μg/mL	Quercetin enhances the efficacy of colistin against colistin resistant kp strains.	>300 µM	(30) (31) (32) (33)
Kaempferol		kale, Spinach, Cabbage, Broccoli, Capers and Onions	16–256 μg/mL	It functions as an adjuvant that counteracts bacterial resistance by blocking efflux pumps and enhancing membrane permeability, thereby enabling effective treatment with lower antibiotic doses.		(34) (35) (36)
Curcumin		Turmeric	128-512μg/ml	When used alongside antibiotics, curcumin improves bacterial eradication against multidrug-resistant (MDR) infections.	59-90 µM	(37) (38) (39) (40)
Baicalein		Chinease Skull-cap	32 – 512 µg/ml	Significantly enhanced antibiotic activities when synergistically used with beta -lactams, tetracycline, and ciprofloxacin.		(41) (42) (43)
Naringin		Citrus fruits	0.5 – 1.0 mg/ml	Naringin acts an anti-inflammatory adjuvant for the treatment of Kp infections.		(44). (45a) (46)
Kuwanon G		Mulberry	8 - 20 µg/ml	Enhanced antimicrobial activity of oxacillin or gentamicin by reducing MIC by 2 - 500 folds against MRSA.		(47) (48) (49, 50)
Apigenin		Parsley, Celery and Chamomile	> 128 µg/ml	_		(51) (52)
Rutin		Buckwheat, Citrus fruits, Berries, Tea, and Capers	256 µg/ml	Rutin reduces MIC value of amikacin by 16 times shows very high synergistic activity.		(53) (54) (55)
Luteolin		Celery, Green peppers, Carrots, Broccoli, and Thyme	31-128 µg/ml	--		(56) (57)
Diadzein		Soybeans	8- 16 µg/ml	In gold nanoparticles formation, daidzein shows synergistic antimicrobial activity against carbapenem resistant kp strains.		(58) (59) (60)
Genistein		Soybeans	Not reported	–		(61)
Catechins		Tea	8- 16 µg/ml	In combination with antibiotics, reduce resistance in MRSA		(62) (63) (64)
Narigenins		Citrus fruits	200- 1024 µg/ml	Naringenin revsrses colistin resistance against multi-drug resistant kp strains.		(65) (66)
Chalcones		Licorice	40-80 µg/ml	:		(67) (68)
Galangin		*Galangal (Ginger family)*	62.5 - 125 µg/ml	Acts as a resistance - modifying agent against bacteria by inhibiting bacterial efflux pumps and increasing membrane permeability, which allows for lower drug doses of antibiotics.		(49) (50) (69) (70)
Eugenol		Clove, Basil and Bay leaf	200 µg/ml	Combination of colistin and eugenol has a significant synergistic antibacterial effect and reverses the sensitivity of colistin resistant Klebsiella strains.		(71) (72) (73)
Geraniin	Terpenes	Geranium	1.25 mg/ml	:		(74) (75)
Linalool		Lavender and coriander (76)	11,250 μg/ml	Along with meropenem MIC reduces to half and act as potential adjuvant for antibiotics against klebsiella infections.		(76) (77)
Terpinen-4-ol		Tea Tree oil	25 mM	-		(78) (79) (80)
Menthol		Mint	2 mg/ml	-		(81) (82)
Citral		Lemongrass	75-150 μg/ml	Interacts synergistically with norfloxacin against MRSA		(83) (84)
Citronellal		Citronella grass, Lemon grass	12.8 mg/ ml	Along with antimicrobial peptides citronellal acts as a adjuvant to kill antibiotic resistant Klebsiella strains		(85) (86) (87)
Camphor		Camphor laurel	57.63 mg/ml	_		(88) (9.)
Carvone		Caraway (89a)	6 - 60 µg/ml	_		(89a) (90) (89b)
Thymol		*Thyme, Ajowan*	475 µg/ml	Thymol and Carvacrol in combination with antibiotics shows synergistic effect against Klebsiella.	1.15 mM	(91) (92.)^93^a)
Carvacrol		*Oregano, Thyme and Savory*	279 µg/ml	Thymol and Carvacrol in combination with antibiotics show synergistic effect against Klebsiella.		(91) (93)
1,8-cineol (eucalyptol)		Eucalyptus	2 - 440 mg/ml	Eucalyptol as a major constituent of essential oils in combination with antibiotics shows enhanced antibacterial properties.	7.31 mM	(94a) (95) (96) (94b)
Myristicin		Nutmeg	0.5 % v/v	_		(97) (98) (9.)
Diterpenes (Ferruginol)		Chamaecyparis lawsoniana	25-250 µg/ml	:		(99) (100)

This table compiles antibacterial plant-derived compounds, detailing their botanical sources and chemical classes. It reports the minimum inhibitory concentration (MIC) values required to inhibit Kp strains. In addition, the table highlights reported synergistic effects of these compounds when used in combination with conventional antibiotics, including their ability to enhance antibiotic efficacy, reduce antibiotic MIC values, and potentially reverse multidrug resistance (MDR). Where available, cytotoxicity data are also provided, expressed as IC values against human cell lines.

Kp virulence has massively disseminated globally in the recent years, although their epidemiology and clinical impact vary considerably across regions. Certain countries, including Israel, Greece, and Colombia, have progressed to endemic transmission (104), whereas others, such as Australia, New Zealand, and Canada, predominantly report imported cases. In regions with a high disease burden, the spread has largely been driven by clonal expansion of Kp, most commonly associated with the globally dominant ST258 lineage (105). In contrast, some countries, including the United Kingdom ST258 lineage, have experienced dissemination primarily through plasmid spread rather than clonal expansion (106). International travel has played a significant role in the introduction of Kp carbapenemase (KPC)-producing strains, particularly through intercontinental movement between the United States and Europe. Further, the emergence of multidrug-resistant (MDR) Kp represents a serious and escalating clinical challenge, particularly in healthcare settings where it frequently causes difficult-to-treat infections due to resistance to multiple antibiotic classes (107).

The management of Kp infections remains a major challenge in clinical infectious disease practice. High mortality rates, coupled with the frequent occurrence of nosocomial transmission, significantly complicate treatment outcomes even in the presence of antibiotic therapy. Current therapeutic approaches against Kp include new-generation β-lactam/β-lactamase inhibitor combinations like ceftazidime-avibactam, meropenem-vaborbactam, and imipenem-relebactam, and cefiderocol (108, 109). Conventional agents such as aminoglycosides, and carbapenems etc are increasingly reserved for combination therapy due to toxicity and resistance concerns (110). In parallel, non-traditional strategies, including bacteriophage therapy, nanoparticle-based drug delivery, and anti-virulence approaches, are under active preclinical and early clinical investigation (111, 112).

Beyond clinical consequences, *Kp* imposes a profound economic and healthcare burden, particularly in low- and middle-income countries, where limited access to effective antimicrobial treatment and weaker healthcare infrastructures contribute to higher morbidity, mortality, and associated costs. Inadequate healthcare infrastructure, limited diagnostic capacity, and shortages of trained healthcare personnel further hinder effective surveillance, timely diagnosis, and appropriate treatment. Together, these economic and systemic constraints enhance the global impact of Kp and highlight the urgent need for improved antimicrobial therapy, affordable therapeutic options, and strengthened healthcare systems worldwide (113).

Unlike previous reviews that primarily address either the epidemiology of antimicrobial resistance or the antibacterial activity of natural products against Kp (110, 114), the present review uniquely integrates the dual antibacterial and immunomodulatory actions of plant-derived compounds with a critical evaluation of their translational limitations. By bridging mechanistic efficacy with clinical feasibility, this work provides a rational framework to guide the development of phytochemicals as adjunctive or alternative therapeutics against multidrug-resistant Kp.

## Dual action of plant-derived compounds against Kp infection

### Direct antibacterial activity

A wide selection of plant-derived compounds has demonstrated significant antibacterial activity against Kp, including MDR. Alkaloids such as *berberine (BBR)* inhibit bacterial growth by intercalating with DNA (115) and disrupting biofilm formation (116), while flavonoids like *rutin* exhibit both antibacterial and antioxidant effects, interfering with membrane integrity and reactive oxygen species (ROS) generation (117). Phenolic compounds and monoterpenes, including *thymol, carvacrol, and 1,8-cineol*, act primarily by disrupting bacterial membranes and inhibiting biofilm formation. *Allyl isothiocyanate* from Brassicaceae plants inhibits key enzymes and flagella-mediated movement in Kp (118, 119). Resinous substances like *propolis*, as well as herbal extracts from species such as Rhazya stricta and Acorus calamus, have also shown strong antibacterial and antibiofilm effects, sometimes targeting specific resistance mechanisms like NDM-1 (120). Traditional herbal formulas combining multiple plant extracts further enhance antibacterial efficacy through synergistic mechanisms (121). Collectively, these phytochemicals act via multiple pathways, including membrane disruption, biofilm inhibition, enzyme interference, and modulation of bacterial gene expression, highlighting their potential as alternative or adjunctive therapies against Kp infections.

### Modulation of inflammatory pathways

Plant-derived natural compounds also inhibit Kp infection by modulating inflammatory pathways. As discussed above, alkaloids like *BBR*, besides exhibiting direct antibacterial activity also regulate the NLRP3 inflammasome (122). It markedly suppressed the expression of NLRP3 inflammasome components in macrophages, thereby limiting M1 polarization and the associated inflammatory response (122).

Flavonoids such as *quercetin and kaempferol* derivatives reduce oxidative stress and suppress TLR4-mediated NF-κB and MAPK activation, thereby attenuating cytokine overproduction (123). Similarly, *curcumin* inhibits the TLR4-MyD88 interaction, preventing NF-κB nuclear translocation (124), while *resveratrol and baicalein* suppress MAPK phosphorylation, thereby ameliorating lung inflammation (125, 126). Other phytochemicals, including *thymol, carvacrol, and 1,8-cineol*, not only exhibit direct antibacterial activity against Kp but also indirectly attenuate inflammation by reducing bacterial load and preventing excessive immune activation (127, 128). Traditional herbal formulas combining multiple plant extracts enhance antibacterial and anti-inflammatory efficacy through synergistic mechanisms. *Naringin (NAR)*, a flavonoid from pummelo peel inhibits NF-κB signaling in alveolar macrophages, reduces neutrophil recruitment, lowers IL-6 and TNF-α, and ameliorates lung inflammation and fibrosis during Kp infection (45b). Andrographis paniculata extracts showed inhibitory activity on growth and biofilm formation of MDR Kp strains along with suppressing AmpC β-lactamase expression (129).

**Figure 1:**
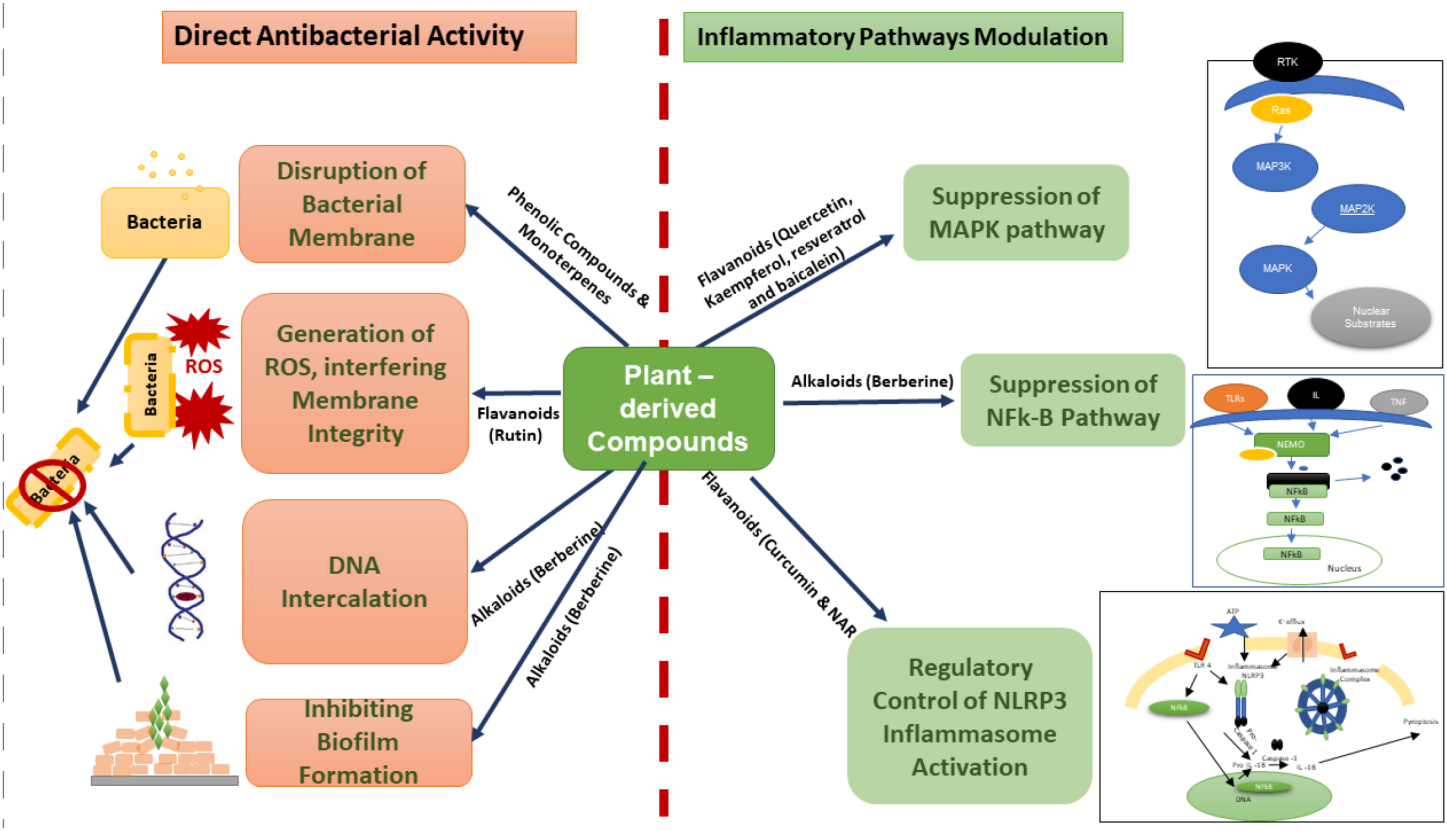
Mechanism of action of plant-derived compounds against Kp. Plant-derived bioactive compounds exhibit a dual mode of action against Kp infection. First, they exert direct antibacterial effects by disrupting bacterial membrane integrity, inducing reactive oxygen species (ROS) generation, inhibiting biofilm formation, and intercalating bacterial DNA. Second, they modulate host inflammatory responses by suppressing MAPK and NF-κB signaling pathways and regulating NLRP3 inflammasome activation, thereby reducing infection-associated inflammation.

The dual therapeutic potential of plant-derived compounds in Kp infections (**Figure 1**) is unequivocal. This integrated action positions phytochemicals as valuable candidates for adjunctive therapy, especially in the context of multidrug-resistant Kp infections (130), where controlling both bacterial growth and host-driven inflammation is critical for clinical outcomes (131).

## Therapeutic implications

The combination of antimicrobial and immunomodulatory effects positions plant-derived compounds as highly valuable candidates in the treatment of Kp infections. Unlike conventional antibiotics that primarily focus on bacterial eradication, many phytochemicals exert a dual mode of action by directly inhibiting bacterial survival and, at the same time, modulating host immune responses to prevent excessive tissue damage 132). This duality is particularly important in the context of Kp, where hypervirulent strains often evade clearance through capsule-mediated resistance and induce overwhelming inflammatory cascades, leading to sepsis and organ dysfunction (9).

Importantly, this dual-action strategy offers a way to counteract the growing threat of multidrug resistance. Since phytochemicals often interfere with bacterial virulence factors such as biofilm formation, exopolysaccharide production, or capsule production, they can weaken pathogen defenses (133) without imposing strong selective pressure that drives resistance. At the same time, their immunomodulatory capacity reduces the severity of inflammation-driven complications (134), such as acute respiratory distress syndrome in severe pneumonia or septic shock during bloodstream infection. This makes them particularly attractive for use in severe and resistant infections where host inflammatory damage is often as dangerous as bacterial burden itself (135).

Furthermore, the integration of phytochemicals with conventional antibiotics holds strong therapeutic promise (136–138). Several studies indicate that natural compounds can restore or enhance antibiotic efficacy, for example, curcumin synergizing with β-lactams or flavonoids potentiating aminoglycoside activity against resistant Kp isolates (117, 139). Such combinations not only improve bacterial clearance but also reduce the required antibiotic dose, thereby limiting toxicity and delaying resistance development. Collectively, these features highlight plant-derived compounds as essential components of next-generation integrative therapies, where their antimicrobial and immunomodulatory effects are harnessed alongside standard antibiotics to achieve fruitful clinical outcomes against Kp infections (140).

## Limitations

Despite the promising antimicrobial and immunomodulatory potential of plant-derived compounds against Kp, several limitations restrict their translation into effective clinical therapies. A major challenge is their poor bioavailability and pharmacokinetics (141); many phytochemicals such as curcumin and quercetin show potent activity *in vitro* but are rapidly metabolized and eliminated *in vivo*, resulting in sub-therapeutic concentrations at the site of infection (142, 143). In addition, variability in plant extracts due to differences in species, growth conditions, harvesting, and extraction methods leads to inconsistent efficacy and reproducibility across studies. The potency of most plant-derived molecules is often weaker compared to standard antibiotics, with minimum inhibitory concentrations (MICs) that are sometimes too high to be clinically achievable without causing toxicity (144, 145). Moreover, while some compounds can inhibit Kp virulence, their action is usually partial and insufficient to inhibit the infection. Lack of standardized formulations (146a) and delivery systems further limits their therapeutic application (147), as many compounds degrade under physiological conditions or require advanced carriers (e.g., nanoparticles, liposomes) to reach effective levels (148, 149). Importantly, the absence of large-scale clinical trials means that safety, efficacy, and optimal dosing remain poorly defined, creating a gap between laboratory evidence and clinical practice (150). Finally, synergistic effects with antibiotics, though promising have not been systematically validated *in vivo*, raising concerns about drug-drug interactions or unpredictable outcomes (151, 152). Collectively, these limitations underscore the need for advanced formulation technologies, rigorous pharmacological testing, and well-designed clinical studies to establish plant-derived compounds as reliable adjuncts or alternatives for managing Kp infections.

These limitations of plant compounds against *Kp or any other infectious agent* can be addressed through *nanotechnology-based delivery systems, standardization, structural modification, and rigorous clinical validation*, making them as potent as well as potential agents against MDR infections.

## Future directions

It is important to investigate how phytochemicals modulate Kp virulence determinants and host immune pathways, including TLR4–NF-κB, MAPK, and inflammasome signaling. Future research on phytochemicals against *Kp* should focus on advancing target-guided studies integrating omics, bioinformatics, and systems-level analyses. Standardization of plant extracts and their formulations remain essential. Future efforts should emphasize reproducible extraction methods, compound purification, and structure–activity relationship (SAR) analyses to improve consistency, potency, and therapeutic predictability (146). In parallel, nanotechnology-based delivery systems such as nanoparticles and liposomal carriers should be systematically explored to overcome poor bioavailability, enhance tissue targeting, and improve pharmacokinetic profiles of promising compounds (153). Robust *in vivo* validation using clinically relevant Kp infection models is required to evaluate antibacterial efficacy, immunomodulatory effects, toxicity, and therapeutic efficacy. In addition, combination therapies comprising phytochemical-antibiotic combinations should be rigorously assessed *in vivo* to confirm efficacy and dosing along with minimizing the risk of adverse drug-drug interactions (154). Collectively, these directions provide a translational roadmap for developing plant-derived compounds as clinically viable therapeutics for the management of Kp infections.

## Conclusion

Accumulating evidence supports the therapeutic relevance of plant-derived compounds in managing Kp infections, particularly those caused by multidrug-resistant strains. These phytochemicals exert multimodal antibacterial effects, including disruption of bacterial membranes, inhibition of biofilm formation, interference with essential enzymatic processes, and modulation of resistance-associated pathways. Concurrently, many compounds act as host-directed immunomodulators, attenuating pathological inflammation through regulation of TLR4–NF-κB, MAPK, and NLRP3 inflammasome signaling, thereby limiting cytokine overproduction and oxidative tissue injury.

This dual antibacterial-immunomodulatory activity supports the use of phytochemicals as adjunctive therapeutics rather than standalone antimicrobials. By reducing bacterial burden while restraining excessive host inflammatory responses, these agents are particularly relevant in severe Kp-associated pneumonia, sepsis, and systemic infections. Representative phytochemicals, including curcumin, berberine, quercetin, resveratrol, and defined plant extracts, consistently demonstrate this integrated activity in preclinical models.

However, clinical translation remains constrained by poor bioavailability, suboptimal pharmacokinetics, and limited human safety data, as most evidence suggests from *in vitro* or *in vivo* studies. Addressing these barriers will require target-based studies, optimized formulation and delivery strategies, and rigorously designed clinical trials. In parallel, systematic evaluation of phytochemical-antibiotic combinations may enhance therapeutic efficacy while mitigating resistance development.

Overall, phytochemicals represent a rational, multi-targeted strategy to combat Kp infection. They do so by simultaneously addressing bacterial virulence and host-driven inflammatory pathology. Strategic integration of mechanistic insight, formulation science, and clinical validation will be essential to advance these agents toward clinically viable interventions against antibiotic-resistant *Kp*.
